# Defining microbiota for developing new probiotics

**DOI:** 10.3402/mehd.v23i0.18579

**Published:** 2012-06-18

**Authors:** Maria Carmen Collado, Christine Bäuerl, Gaspar Pérez-Martínez

**Affiliations:** Institute of Agrochemistry and Food Science, Spanish National Research Council (IATA-CSIC), Department of Biotechnology, Unit of Lactic Acid Bacteria and Probiotics, Paterna, Valencia, Spain

**Keywords:** microbiota, probiotic, diet, health

## Abstract

The human body harbors complex communities of microbes that play a prominent role in human health. Detailed characterization of the microbiota in the target population forms the basis of probiotic use. Probiotics are defined as live bacterial preparations with clinically documented health effects in humans, and independent of their genus and species, probiotic strains are unique and their beneficial properties on human health have to be assessed in a case-by-case manner. Understanding the mechanisms by which probiotics influence microbiota would facilitate the use of probiotics for both dietary management and reduction in risk of specific diseases. The development of high throughput sequencing methods has allowed metagenomic approaches to study the human microbiome. These efforts are starting to generate an inventory of bacterial taxons and functional features bound to particular health or disease status that allow inferring aspects of the microbiome's function. In the future, this information will allow the rational design of dietary interventions aimed to improve consumer's health via modulation of the microbiota.

The human microbiota (HM) is a complex system of many microbial communities inhabiting a diversity of environmental niches throughout the human body. HM exhibits large variation among individuals in relation to internal and external factors, such as genetic factors, age, diet, and health, and remains in a complex equilibrium. Although the exact composition of the microbiota is not known, advances in genomic technologies have recently begun to unravel our microbial partners. It is known that about 75% of the gut microbiota is covered by already known and dominant phyla (Actinobacteria, Firmicutes, and Bacteroidetes), while a fraction of about 25% is still unknown. It is estimated that each individual houses a consortium of 1,000–1,150 prevalent bacterial species (1) whose collective genome (microbiome) contains at least 100 times as many genes as the human genome.

The development of the human gut microbiota is a complex process that has been traditionally assumed to start at birth, although recent reports indicate that microbial colonization may begin earlier as bacteria have been detected in meconium, umbilical cord, and amniotic fluid (2–5). In infants, microbiota development is a fast process that depends on the first inoculi received from the environment, and also the maternal microbiota, mode of delivery, type of feeding, including weaning food practices and the use of antimicrobials (6). The early GIT microbiota is often dominated by one or a few genera and among breast-fed infants, *Bifidobacterium*-dominated microbiotae are more frequent than among infants fed with formula, but other compositions are also common. A large shift in microbiota composition accompanies the introduction of solid foods into the diet (7–10). It is likely that the beneficial impact achieved for the infant with breastfeeding is a combination of a balanced supply of nutrients, bioactive proteins, and indigestible oligosaccharides, as well as bifidogenic bacteria in breast milk (11). Compilations of long-term studies have shown that breast-fed infants have lower risk of diabetes (12), hypercholesterolemia (13), cardiovascular disease (14), and obesity (15) in adulthood than formula-fed infants, although the causality is difficult to ascertain.

## Human microbiota composition and health

In recent years, the increase in microbiota-related research has provided important advances toward establishing the identity of specific microbes and microbial groups or microbial molecules contributing to various aspects of host physiology and health. Studies on human microbiota should include microbial ecology and analysis of the complex metabolism of the microbial community, as well as various host–microbial interactions occurring at the interface between microbes and host intestinal epithelia. Such studies should lead to an understanding of the impact of the microbiota on human health and disease. Concurrently, host factors involved in various aspects of development and maturation targeted by the microbiota have been identified.

A balance among microbial groups present in the human gut is crucial for maintaining health. When this balance is disturbed, the host–microbe relationship can progress toward a disease state. Altered intestinal colonization by commensal microorganisms as well as high interindividual variability and reduced microbial diversity have been reported in preterm infants (16, 17), increasing the risk to develop later disease. Several gastrointestinal pathologies such as irritable bowel diseases (IBD) or syndromes (IBS), necrotizing enterocolitis (NEC), obesity, various forms of colitis, and even autism have been linked to disturbances in microbiota or alterations of the intimate cross-talk between these microbes and human cells (18). Numerous studies have also linked early gut microbiota to the development of atopic diseases, but no specific microbes have yet been identified with consistently harmful or protective roles regarding atopy (19–21). However, some reports have suggested that the gut microbiota could regulate host energy homeostasis and adiposity, as differences in microbial composition can explain an increased capacity of the obesity-associated microbiome to harvest energy from the diet (22, 23). Other studies have examined gut microbiota composition in human obesity and type-2 diabetes and the impact of weight reduction on microbiota (24–26). All these works suggest that there could be a link between gut microbiota composition and host's health. Further studies will confirm if the rationale for modulating the gut microbiota by means of probiotics could also derive in health improvement.

## Challenges for probiotic development based on microbiota research

There is a growing interest in studying beneficial microbes from the human microbiota with specific functions, which could eventually be used as probiotics in foods or supplements to improve human health and prevent or treat diseases. According to the FAO–WHO definition of probiotics – ‘live microorganism which when administered in adequate amounts confers a health benefit on the host’ (27) and food regulations that are currently in force, those beneficial effects must be scientifically demonstrated. It is important to underline that probiotic strains are unique, limiting the extrapolation of results from one strain to another. It is well-known that different bacterial strains of the same genus and species may exert completely different effects on the host. Therefore, the specific properties and characteristics of individual strains should be well defined and the effect on health of each strain should be demonstrated in a case by case manner. Then, the selection of potential probiotic strains from appropriate sources depending on the target population constitutes a promising approach. Some clear challenges have been identified through this study.

### Novel uses and applications of probiotics

In general, any disorder in which an aberrant microbiota or an unappropriate immune response may play a role are potential targets for probiotic intervention, even though they may not take place at gut level. Studies have shown that administration of probiotics to pregnant women, nursing mothers, or newborns can influence the establishment and composition of infant gut microbiota (28–30), impacting early and later in life. Probiotic bacteria have been usually used to treat and prevent some gastrointestinal disturbances such as IBD, IBS, or diarrhea, and new evidences support the use of probiotics in the prevention and treatment of a number of diseases including atopic diseases, immune disorders, obesity, and diabetes, although new extraintestinal applications are the getting interest of industry and consumers. Disturbances IN microbiota have been identified in other intestinal disorders, including diverticulitis and extraintestinal conditions, such as elderly people suffering severe frailty. Further, patients with severe systemic inflammatory response syndrome showed lower levels of bifidobacteria and lactobacilli and higher levels of pathogenic microorganisms than healthy subjects. Reduced levels of bifidobacteria have also been shown in multiple sclerosis patients. In addition, the current evidence for a role of bacteria (commensals, probiotics, and pathogens) as key modulators of gut–brain communication (31) suggests the potential role of probiotics on the gut–brain axis. Although, so far, probiotics have not been tested in these settings, these studies indicate potential targets for the future development of probiotic products.

### Study of other gut microbiota components as probiotic

The complexity of the gut microbiota provides a very promising source of new probiotic organisms, and in many research works, gut immunologists prefer to use the terms ‘commensal bacteria’, instead. Enterocytes and dendritic cells in the gut mucosa can discriminate pathogenic from commensal bacteria, through specialized receptors and signal transduction pathways crucial for maintaining intestinal immune homeostasis and mechanisms of innate defense (32, 33). These cascades of molecular signals are nowadays only partially defined and constitute the basis of the demonstrated immunomodulatory effect of these bacteria. In this regard, other bacteria than those commercially used as probiotics have been scarcely studied. Different microorganisms are used as human probiotics, the most commonly used probiotics are intestinal strains of *Lactobacillus*, *Bifidobacterium*, and *Enterococcus* species, for which technology has been developed for their industrial production. However, other intestinal microbes may also have a beneficial role in human health. *Escherichia coli* is among the first colonizers of the infant gut, and although this species harbor pathogenic strains, the *E. coli* probiotic strain Nissle 1917 has been found to reduce the number and incidence of infections, to stimulate specific humoral and cellular responses, and to induce the non-specific natural immunity in infants (34, 35). In addition to commercially used *Saccharomyces boulardii*, which has been reviewed extensively, a number of spore-forming bacilli have been claimed to show probiotic effects (36, 37). In fact, *Bacillus subtilis* strains have also been used commercially as their spores offer great manipulation and packaging advantages over other bacterial species (38). Other *Bacillus* strains have been studied such as *Bacillus cereus* var. *toyoi* (39) and *Bacillus clausii* (40). Furthermore, other gram positive bacteria have also been solidly claimed to have great probiotic potential. Different studies attribute immunomodulatory properties to strains of *Propionibacterium freudenreichii* used individually (41) or in combination with lactic acid bacteria and bifidobacteria in intervention trials (42). Further, humans lack the enzymes needed to metabolize oxalate, a toxic compound causing hyperoxaluria and calcium oxalate urolithiasis. This compound in humans can be eliminated through excretion in urine, forming insoluble calcium oxalate and elimination in feces, or oxalate degradation by microbiota. *Oxalobacter formigenes* and *Lactobacillus* and *Bifidobacterium* species are the best studied in this regard, with oxalate degradation in the lactic acid bacteria being both species- and strain-specific (43). Recently, a *Bacteroides* strain, closely related to *Bacteroides dorei*, able to reduce cholesterol was isolated from the gut microbiota of a subject with a high ability to reduce cholesterol to coprostanol (44).

### Potential of probiotics to modulate microbiota

One of the major challenges found, from early research on microbiota composition, is to define the composition and complete functionality of the *normal microbiota* in healthy individuals. Recent studies have shown differences in the microbiota composition of healthy subjects from different locations (45–47). Could probiotic intervention strategies be developed targeted to restore this normal population profiles in cases of aberrant microbiota associated to diseases? Different research publications until present indicate that administration of *Lactobacillus* and *Bifidobacterium* probiotics did not affect the overall populations proportions in the gut microbiota; however, a significant health effect was observed, and in all cases, strains administered dominated the respective groups in the gut (48–51). A deeper understanding on the microbiome-modulating abilities of specific probiotic strains is needed and, so far, the metagenomic data available from human intervention studies with probiotics and their impact on the microbiome are limited.

## Conclusions

Knowledge of intestinal microbiota development, nutrition, immunity, and specific diseases should be carefully combined with information of the genome of potential probiotic strains to find new probiotics with disease risk-modifying properties.
